# Comprehensive analysis of autophagy-related clusters and individual risk model for immunotherapy response prediction in gastric cancer

**DOI:** 10.3389/fonc.2023.1105778

**Published:** 2023-03-03

**Authors:** Yanxin Yao, Xin Hu, Junfu Ma, Liuxing Wu, Ye Tian, Kexin Chen, Ben Liu

**Affiliations:** Department of Epidemiology and Biostatistics, Key Laboratory of Molecular Cancer Epidemiology of Tianjin, National Clinical Research Center for Cancer, Tianjin Medical University Cancer Institute and Hospital, Tianjin Medical University, Tianjin, China

**Keywords:** gastric cancer, autophagy-related genes, immunotherapy, prognostic signature, oxidative stress

## Abstract

**Introduction:**

Autophagy can be triggered by oxidative stress and is a double-edged sword involved in the progression of multiple malignancies. However, the precise roles of autophagy on immune response in gastric cancer (GC) remain clarified.

**Methods:**

We endeavor to explore the novel autophagy-related clusters and develop a multi-gene signature for predicting the prognosis and the response to immunotherapy in GC. A total of 1505 patients from eight GC cohorts were categorized into two subtypes using consensus clustering. We compare the differences between clusters by the multi-omics approach. Cox and LASSO regression models were used to construct the prognostic signature.

**Results:**

Two distinct clusters were identified. Compared with cluster 2, the patients in cluster 1 have favorable survival outcomes and lower scores for epithelial-mesenchymal transition (EMT). The two subtypes are further characterized by high heterogeneity concerning immune cell infiltration, somatic mutation pattern, and pathway activity by gene set enrichment analysis (GSEA). We obtained 21 autophagy-related differential expression genes (DEGs), in which PTK6 amplification and BCL2/CDKN2A deletion were highly prevalent. The four-gene (PEA15, HSPB8, BNIP3, and GABARAPL1) risk signature was further constructed with good predictive performance and validated in 3 independent datasets including our local Tianjin cohort. The risk score was proved to be independent prognostic factor. A prognostic nomogram showed robust validity of GC survival. The risk score was significantly associated with immune cell infiltration status, tumor mutation burden (TMB), microsatellite instability (MSI), and immune checkpoint molecules. Furthermore, the model was efficient for predicting the response to tumor-targeted agent and immunotherapy and verified by the IMvigor210 cohort. This model is also capable of discriminating between low and high-risk patients receiving chemotherapy.

**Conclusion:**

Altogether, our exploratory research on the landscape of autophagy-related patterns may shed light on individualized therapies and prognosis in GC.

## Introduction

Being the fifth most common cancer globally, GC has been one of the leading causes of cancer death (1). In 2018, the mortality rate of gastric cancer was 8.2% because it is usually at an advanced stage when diagnosed (2). Surgery, chemotherapy, and chemo-radiotherapy are effective methods for gastric cancer (3, 4). Currently, molecular targeted therapy and immunotherapy are also increasingly highlighted (5). Lauren classification and WHO classification, the current histological classification methods of GC, sometimes affect the choice of endoscopy or surgery. Still, they are not enough to guide the proper treatment of individual patients (6, 7). GC is classified into four different molecular subtypes in Cancer Genome Atlas (TCGA) (8), among four molecular subtypes, Epstein-Barr virus-positive (EBV+) and microsatellite instable (MSI) subtypes play a certain role in the prognosis and treatment response of GC. For drug therapy, MSI/dMMR status and HER2 (9, 10) are strongly predictive biomarkers rather than molecular subtypes. Even so, the first-line drug targets HER2 receptor shows limited utility against GC (11). The high heterogeneity of gastric cancer has hindered the development of its treatment. Exploring new pathways and targets for molecular targeted therapy remains a trend in the molecular treatment of patients with GC.

Autophagy is a form of programmed cell death (12), playing a crucial role in the initiation and development of GC (13). Closely linked to the effect of tumors, autophagy responds to different stress, such as nutrient deprivation, hypoxia, and various cytotoxic insults (14). Oxidative stress is caused by the continuous elevation of reactive oxygen species(ROS), and ROS formation is essential for autophagy (15). When the tumor is in the stage of initiation and early steps of progression, autophagy suppresses tumor progression. However, autophagy always promotes tumor survival in advanced cancers, as autophagy helps tumors deal with a hard environment like hypoxia (16). Autophagy is bidirectionally related to immune checkpoint molecules (17, 18), EMT (19), MSI (20), and TMB (21), credible immunotherapy biomarkers for cancers (22, 23). Thus, it is a double-edged sword in the immunotherapy of GC. Autophagy provides targets of immunotherapy for regulating immune response by influencing cells and the release of cytokines. Some research has shown that conventional cancer treatment, including immunotherapy, combined with autophagy-based inducer or inhibitor, may be more effective (24). The mechanism of autophagy in the development of GC is relatively clear. However, due to the double-sided effect of autophagy, the role of autophagy in the prognosis of patients with GC still needs further exploration. It is necessary to analyze the function of autophagy-related genes in gastric cancer and its contribution to prognosis.

In the present study, we collected autophagy-related genes and divided patients with GC from TCGA and GEO database into two subtypes. A prognosis signature of gastric cancer patients has been established with four prognosis-related genes selected from 21 differentially expressed genes (DEGs).

## Materials and methods

### Gastric cancer dataset source and preprocessing

The RNA-Seq profile and clinical information of GC patients were downloaded from the TCGA database (http://www.cancergenome.nih.gov/), and the Gene Expression Omnibus (GEO)(RRID : SCR_005012) database (https://www.ncbi.nlm.nih.gov/geo/). Tianjin cohort, which included 90 samples, served as the validation set (25, 26). Batch effects of 6 cohorts enrolled were removed by sva R package. Sample sizes were not chosen using power analysis, as effect sizes could not be pre-determined.

### DEGs screening

Consensus clustering analysis was used to classify GC patients into subtypes carrying out by the Consensus ClusterPlus R package. Differentially expressed genes (DEGs) between different subtypes were selected by limma R package(LIMMA, RRID : SCR_010943), with|log2 fold change (FC)|>0.5, and P<0.05.

### Construction of prognostic signature and risk score calculation

Univariate Cox regression analysis and LASSO regression were used based on selected DEGs, and P<0.05 was considered confident in statistics. The risk score was built by selected prognostic genes, calculated as follows:


$$riskscore=\sum_{i=1}^{n}Coef_{i}\times X_{I}$$

this equation, the Coef means the gene coefficient, and X means the gene expression level. All sample enrolled in this study was divided into high-risk and low-risk groups by the median risk score value.

### Analysis of subtypes biological features and evaluation of prognostic signature

Details were available in Supplements.

### Statistical analysis

R software 4.1.0 was applied to conduct all statistical analyses of this study. The Pearson correlation test analyzed the correlation between molecules. Verified Two-tailed p<0.05 was regarded statistically significant. The analysis required no randomization or blinding.

## Results

### Two clusters are identified based on autophagy-related genes

**Figure 1:**
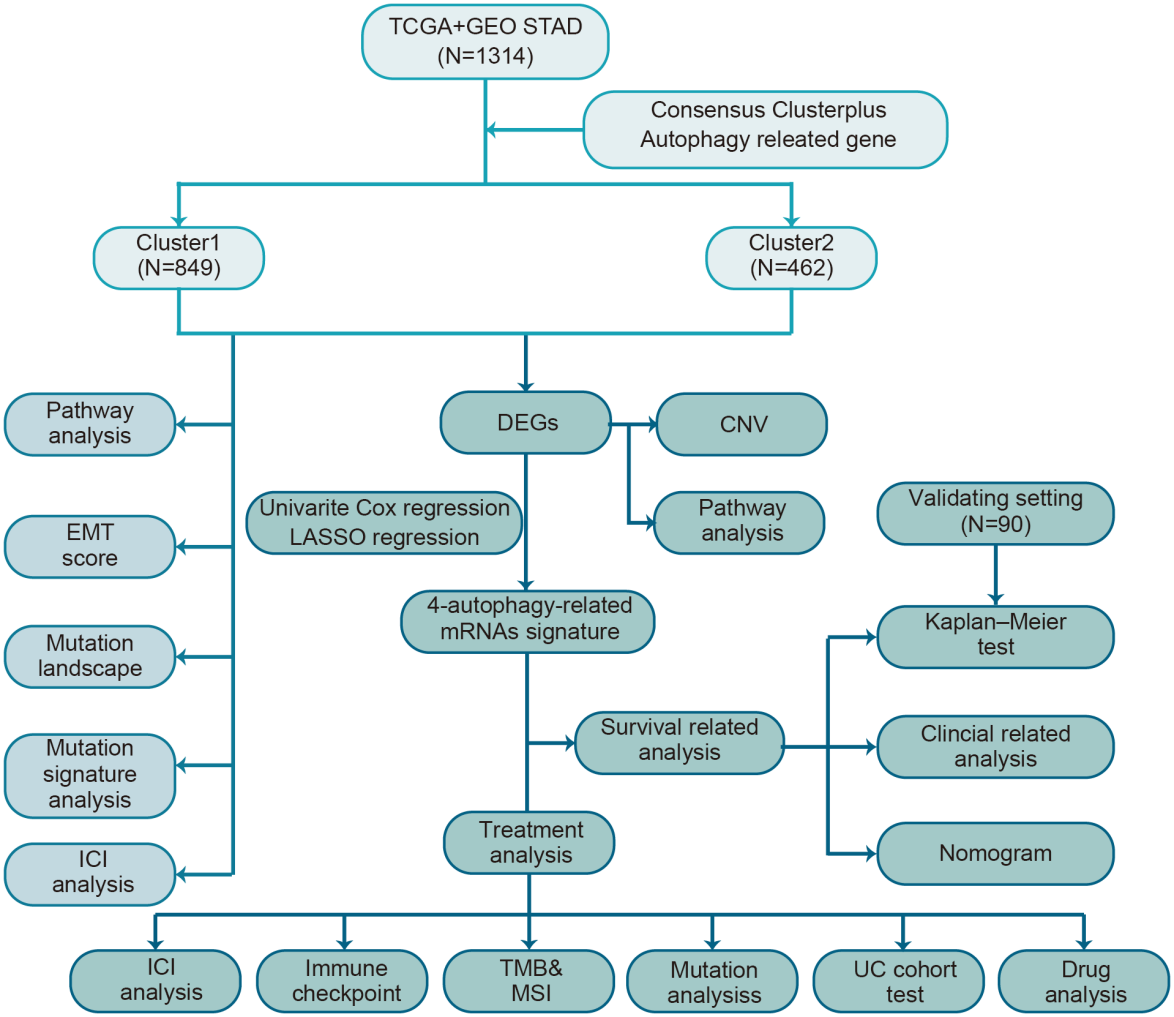
The flow chart of the study design for autophagy-related subtyping and risk model construction.

**Figure 2:**
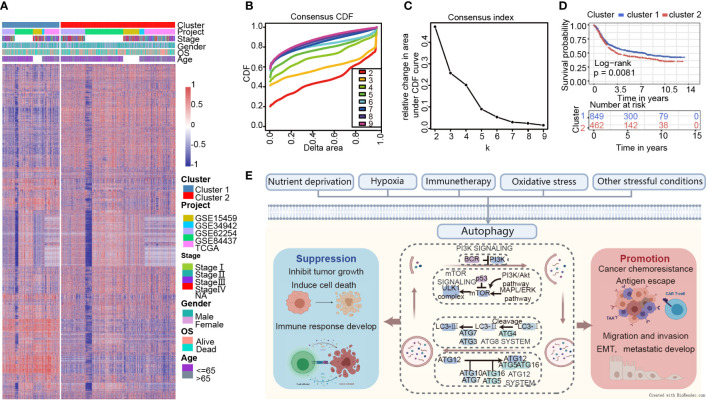
Consensus clustering indicates that 2 clusters are the greatest number of stable clusters across clustering techniques. **(A)** Heatmap of gene expression with the project, stage, gender, OS, and age between cluster 1 and 2. **(B)** Consensus clustering cumulative distribution function (CDF) for k = 2 to k = 9. **(C)** Relative change in area under CDF curve according to various k values. **(D)** Survival analysis of Cluster 1 and Cluster 2 in the training set. **(E)** Schematic diagram for the molecular machinery of autophagy and the dual role of autophagy in cancer progression.

A brief flow chart showed the process of contradistinction between two clusters and building prognostic signature (**Figure 1**). The batch effect among all cohorts was removed before analysis (**Figures S1A, B**). A total of 1401 gastric cancer samples were enrolled in this analysis. Among them, 1311 GC patients from TCGA and GEO were used as a training set. Moreover, 90 patients from the Tianjin cohort were used as a validation set. A total of 222 autophagy-related genes were acquired. Only genes common to all studies were included. The clinical information of the six cohorts enrolled (**Table S1**). According to the expression of autophagy-related genes, we performed a consensus clustering analysis of GC patients from the training set, and optimal k was 2, which was identified from the range between 2 and 9 with optimal clustering stability (**Figures 2B, C**, **S2A, B**). The Consensus clustering results suggested different gene expression patterns in two clusters (**Figure 2A**). All training set samples were divided into cluster 1(n=849) and cluster 2(n=462). Kaplan–Meier survival curves showed the OS was significantly poorer in cluster 1 than those in cluster 2(p = 0.0081, HR = 1.2445, 95% CI: 1.0580 1.4637), (**Figure 2D**). A schematic diagram was applied to briefly show autophagy’s dual role in cancer progression and immunotherapy (**Figure 2E**)

### The characteristics are quite distinct between the two clusters

**Figure 3:**
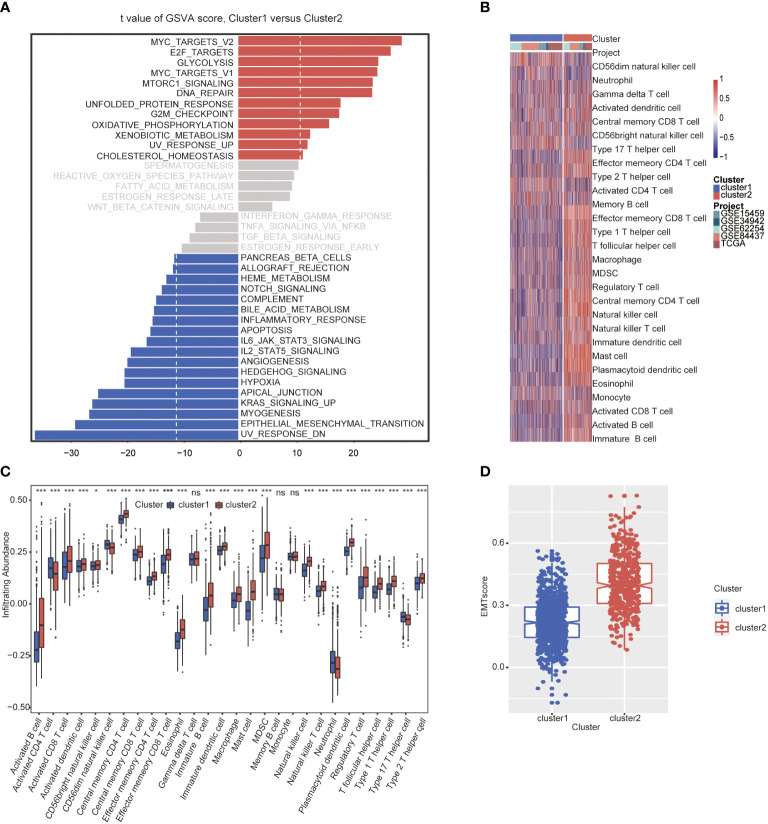
Molecular and immune characteristic analysis between two autophagy subtypes. **(A)** Differential expression of genes in GC patients between cluster 1 and cluster 2 was analyzed by GSVA. **(B)** Heatmap of the two clusters based on autophagy-related genes expression for 21 immune terms. **(C)** Box plots to visualize significantly different immune cells between two clusters. **(D)** Box plots shows that EMT score of cluster 1 is higher than cluster 2 (P<0.001) (*P< 0.05; ***P< 0.001; NS or ns, P > 0.05).

**Figure 4:**
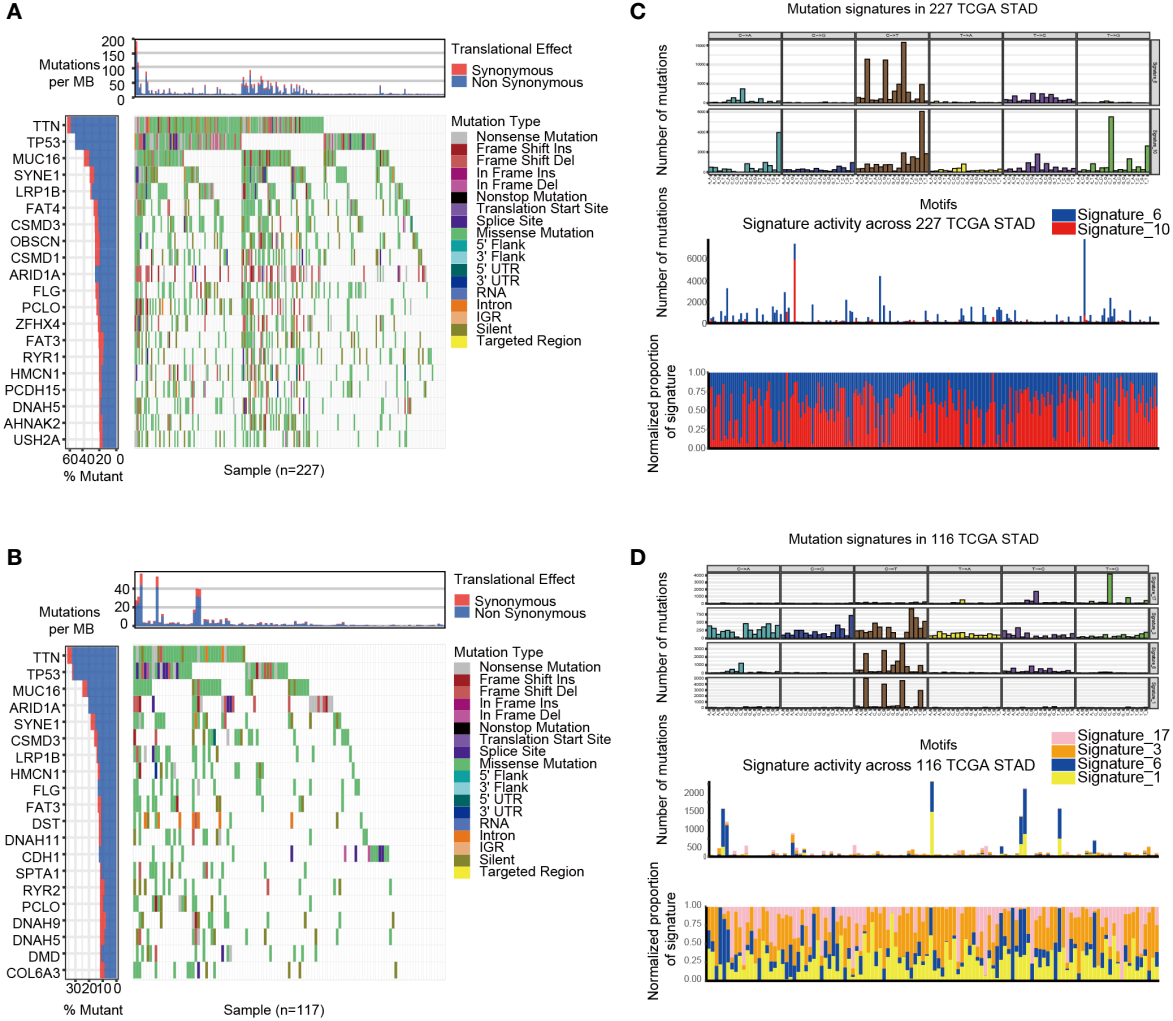
The mutation landscapes of cluster 1 and cluster 2. **(A-B)** The waterfall plot illustrates the single most damaging variant found per gene and per sample in TCGA, with colors indicating mutation types of cluster 1**(A)** and cluster 2 **(B)**. **(C)** Mutation signature of cluster 1 is focused on signature 6 and 10. **(D)** Mutation signature of cluster 2 is focused on signature17, 3, 6 and 1.

GSVA analysis was conducted to explore the characteristics between cluster 1 and cluster 2. The results showed two clusters enriched in differentially expressed pathways: cluster 1 enriched in MYC target gene sets (MYC_TARGETS_V2), E2F target gene sets(E2F_TARGETS) which are related with cell circle, and Gene sets involved in glycolysis and gluconeogenesis (GLYCOLYSIS), cluster 2 enriched in Gene sets down-regulated in response to ultraviolet (UV) radiation(UV_RESPONSE_DN), Gene sets defining epithelial-mesenchymal transition (EPITHELIAL_MESENCHYMAL_ TRANSITION), and gene sets involved in the development of skeletal muscle (MYOGENESIS)(**Figure 3A**). A total of 28 kinds of immune cells were quantified between two clusters, such as B cells, T cells, NK cells, and macrophages (27). And a heatmap was drawn with the project of samples. Heatmap showed that the immune cell infiltration level of cluster 1 was lower than cluster 2 (**Figure 3B**). Further analysis revealed that most of the 28 immune-related terms between cluster 1 and cluster 2 were very highly significant differences (*P*<0.001) (**Figure 3C**). The EMT scores, which estimated the degree of epithelial-mesenchymal transition, had statistically significant differences between the two clusters(P<0.001) (**Figure 3D**). The different frequency and types of mutations in genes between two clusters were shown in these waterfall plots. Somatic mutations analysis suggested that the TTN, TP53, and MUC16 are the highest mutation frequency both in cluster 1 and cluster 2. Though the top three of the mutant genes in the two clusters were the same, the fourth and fifth ones were not inconsistent: cluster 1 are SYNE1 and LRP18, as cluster 2 are ARID1A and SYNE1(**Figures 4A, B**). Next, the COSMIC mutation signature analysis showed two clusters associated with defective DNA mismatch repair (signature 6). The results implied that mutation of cluster 1 was associated with recurrent POLE somatic mutations (signature 10), and cluster 2 was related to the failure of DNA double-strand break-repair and endogenous mutational process (signature 17, signature 3 and signature 1) (**Figures 4C, D**).

### Twenty-one autophagy-related DEGs are selected, and the four-gene prognostic signature is constructed

**Figure 5:**
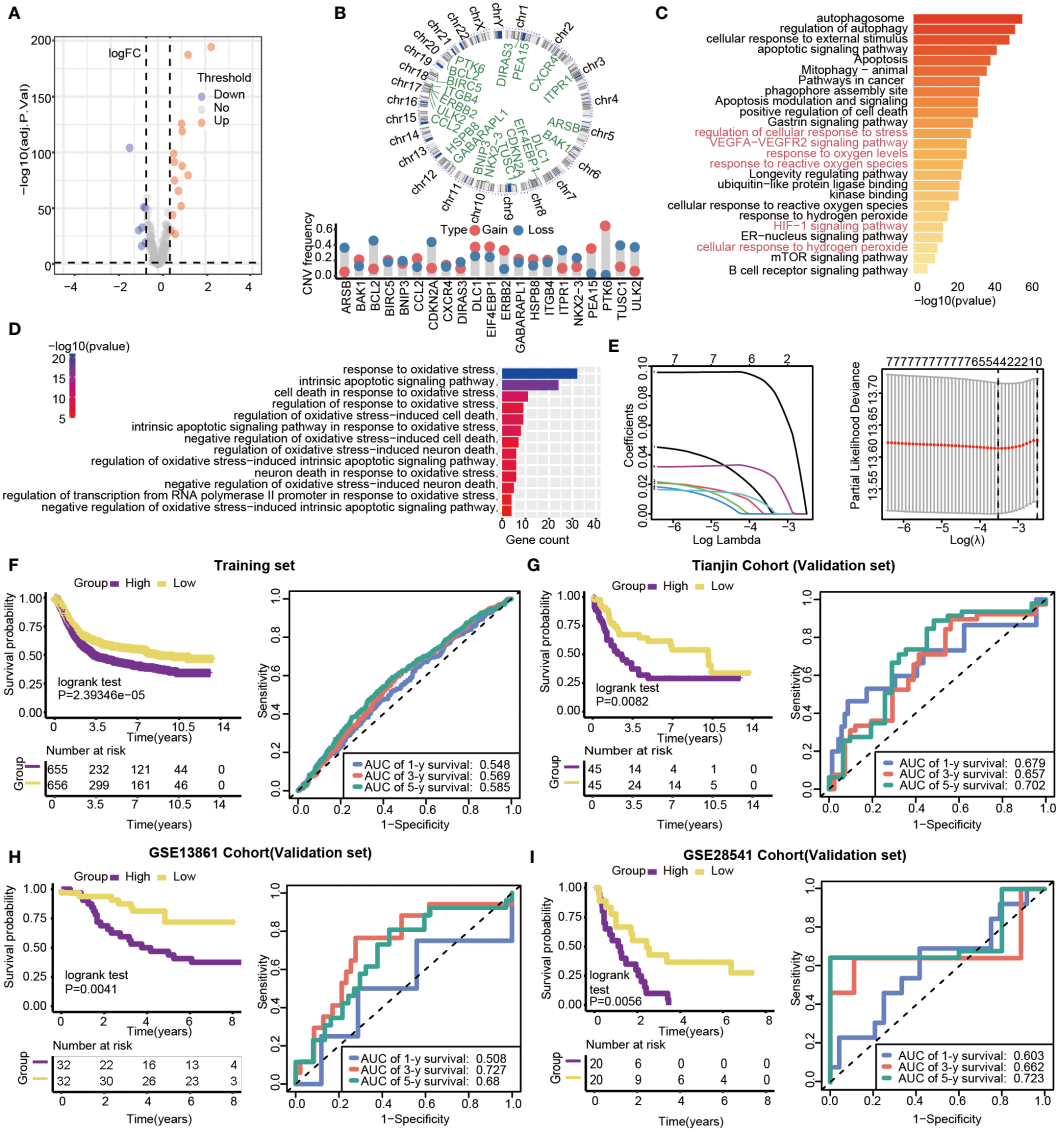
Gene features of DEGs and survival analysis based on the risk model in training and validation datasets. **(A)** Volcano plot of differential gene expression analysis. **(B)** Circular map of CNV regions in DEGs(top), and CNV frequency of 21 DEGs(bottom). **(C)** Genetic functional enrichment analysis, and the pathways related to oxidative stress are colored red. **(D)** GO enrichment analysis of oxidative stress-related pathways. **(E)** LASSO deviance profiles, and LASSO coefficient profiles. **(F–I)** Kaplan-Meier survival curves and 1-,3- and 5-year ROC curves for patients between high- and low-risk groups in the training set (n=1311, P<0.05), Tianjin validation set (n=90, P<0.05), GSE13861validation set (n=64, P=0.0041) and GSE28541 validation set (n=40, P=0.0056).

With limma R package, 203 genes were differentially expressed, and 21 DEGs (|log 2 FC|>0.5, *P*<0.05) were selected from all autophagy-related genes, including 14 up-regulated and 7 down-regulated genes (**Figure 5A**). The results showed that all of the 21 DEGs had CNV alteration, including both copy number amplifications (PTK6, DLC1and EIF4EBP1, etc.) and losses (BCL2, CDKN2A, and TUSC1, etc.) (**Figure 5B**). By pathway enrichment analysis of 203 differential genes through Metascape, autophagy, oxidative stress, and lifespan-related pathways were significantly enriched between subtypes (**Figure 5C**). Interestingly, the results indicated that pathways related to oxidative stress were highly enriched, such as response to oxidative stress, intrinsic apoptotic signaling pathway, and cell death in response to oxidative stress (**Figure 5D**).

Prognosis-related genes were selected by Univariate Cox regression and LASSO regression to constitute prognosis signature (**Figure 5E**). The risk score was calculated as follows:


$$riskscore=0.0877\times exp_{PEA15}+0.0275\times exp_{HSPB8}+0.0053\times exp_{BNIP3}+0.0053\times exp_{GABARAPL1}$$

We calculated the 4-autophagy-related genes signature risk score for each patient in the training set and ranked them with their risk scores. Then, patients were divided into high- (n=655) and low-risk group(n=656) by the median risk score (**Table S2**). Patients in the low-risk group had significantly longer OS than those in the high-risk group (log-rank test *P*=2.39346×10^-5^)(**Figure 5F**, left panel). A time-dependent ROC curve was used to predicting the 1-, 3-, 5‐year survival. The area under the curve (AUC) value of the ROC curve reflected the quality of the ROC curve. In the training set, 1-year AUC = 0.548, 3-year AUC = 0.569, and 5-year AUC = 0.585 (**Figure 5F**, right panel). In the external data set, the distribution of survival time indicated that the high-risk group had a better prognosis. The result of K-M survival analysis indicated OS was shorter in the high-risk group than in the low-risk group (log-rank test *P*= 0.0082) (**Figure 5G**, left panel). In the validation set, the 1‐year AUC was 0.679, the 3‐year AUC was 0.657, and the 5-year AUG was 0.702(**Figure 5G**, right panel). Beyond this, two external validations datasets (GSE13861 and GSE28541) were used to verify our method. The two external validation results suggested that patients identified as high risk had a poorer prognosis than patients identified as low risk (GSE13861, P =0.0041; GSE28541, P = 0.0056) (**Figure 5H**, left panel, **Figure 5I**, left panel). Furthermore, the 5-year ROC curve was 0.68 and 0.723, respectively (**Figure 5H**, right panel, **Figure 5I**, right panel).

### The constructed risk score is an independent prognosis factor for GC

**Figure 6:**
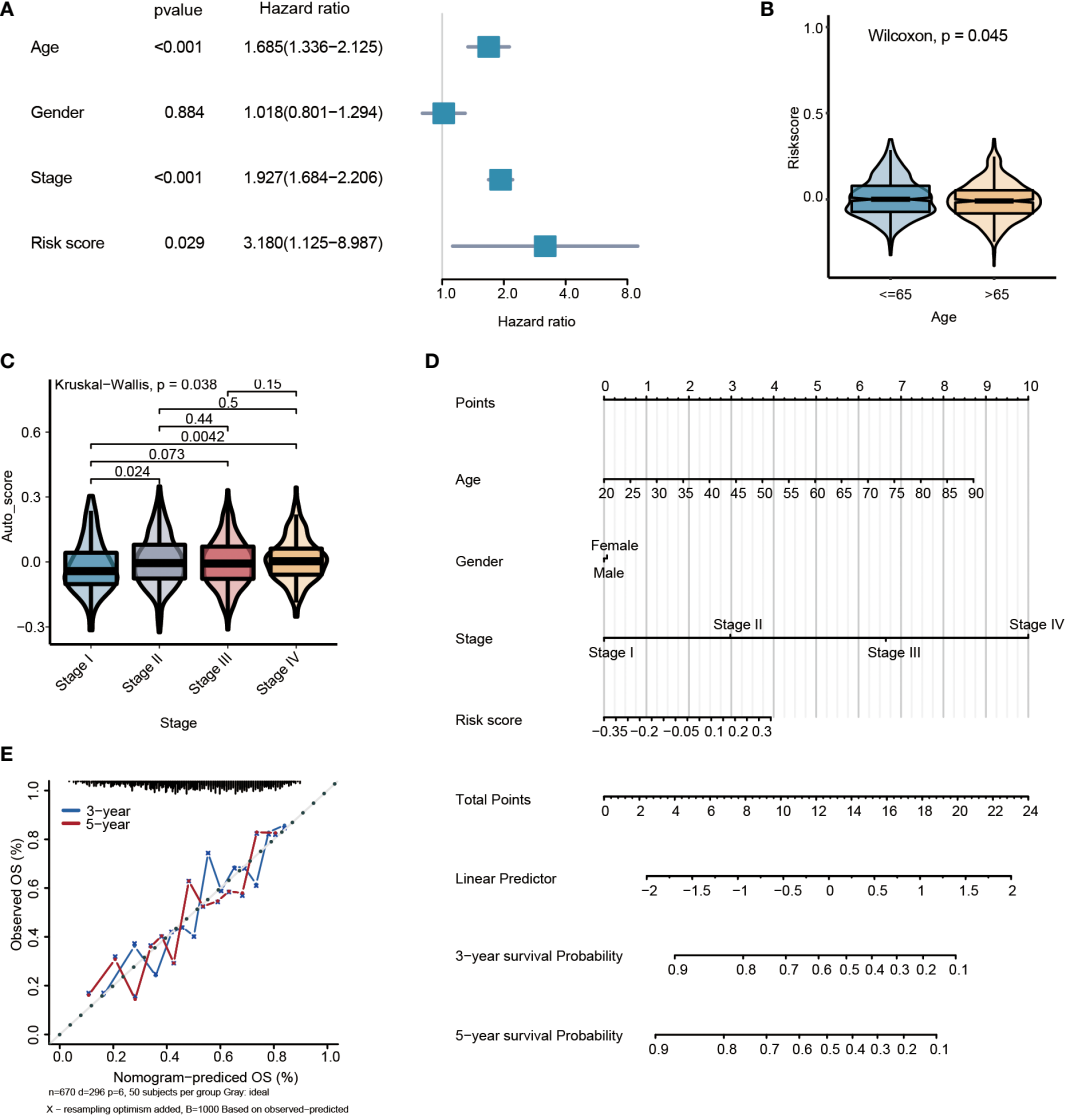
Multivariate survival analysis and nomogram construction for predicting overall survival combining the risk signature with clinicopathological characteristics. **(A)** Forest plot of hazard ratios from the multivariable Cox proportional hazard regression model suggests that risk score is an independent risk factor. **(B)** Box plot of risk score levels grouped by age (P<0.05). **(C)** Box plot of risk score levels grouped by stage (P<0.05). **(D)** Nomogram predicting overall survival probability for GC patients. **(E)** Calibration of the nomogram for 3- and 5-year.

By multivariate Cox regression analysis, the risk score was the independent prognosis factor for gastric cancer (*P*=0.029, HR=3.180, 95%CI 1.125~8.987) (**Figure 6A**). The risk score was significantly associated with clinicopathological factors, including age (Wilcoxon test, *P*= 0.045) and stage (Wilcoxon test, *P*=0.038) (**Figures 6B, C**). Nomogram, which calculated scores for every GC patient, was applied to estimate the prognosis of gastric cancer patients visually and accurately. The nomogram was established using the training set, which can predict 3- and 5-year OS based on the multivariate Cox regression model (**Figure 6D**). The risk score, age, gender, and stage were parameters included in the nomogram. The concordance index for the nomogram was 0.6771(95%CI, 0.6456 to 0.7088). The calibration curves of 3- and5-year prediction showed good consistency compared with the ideal model, indicating that the nomogram was stable in the prognosis of GC patients (**Figure 6E**).

### The low-risk group is more suitable to receive immunotherapy

**Figure 7:**
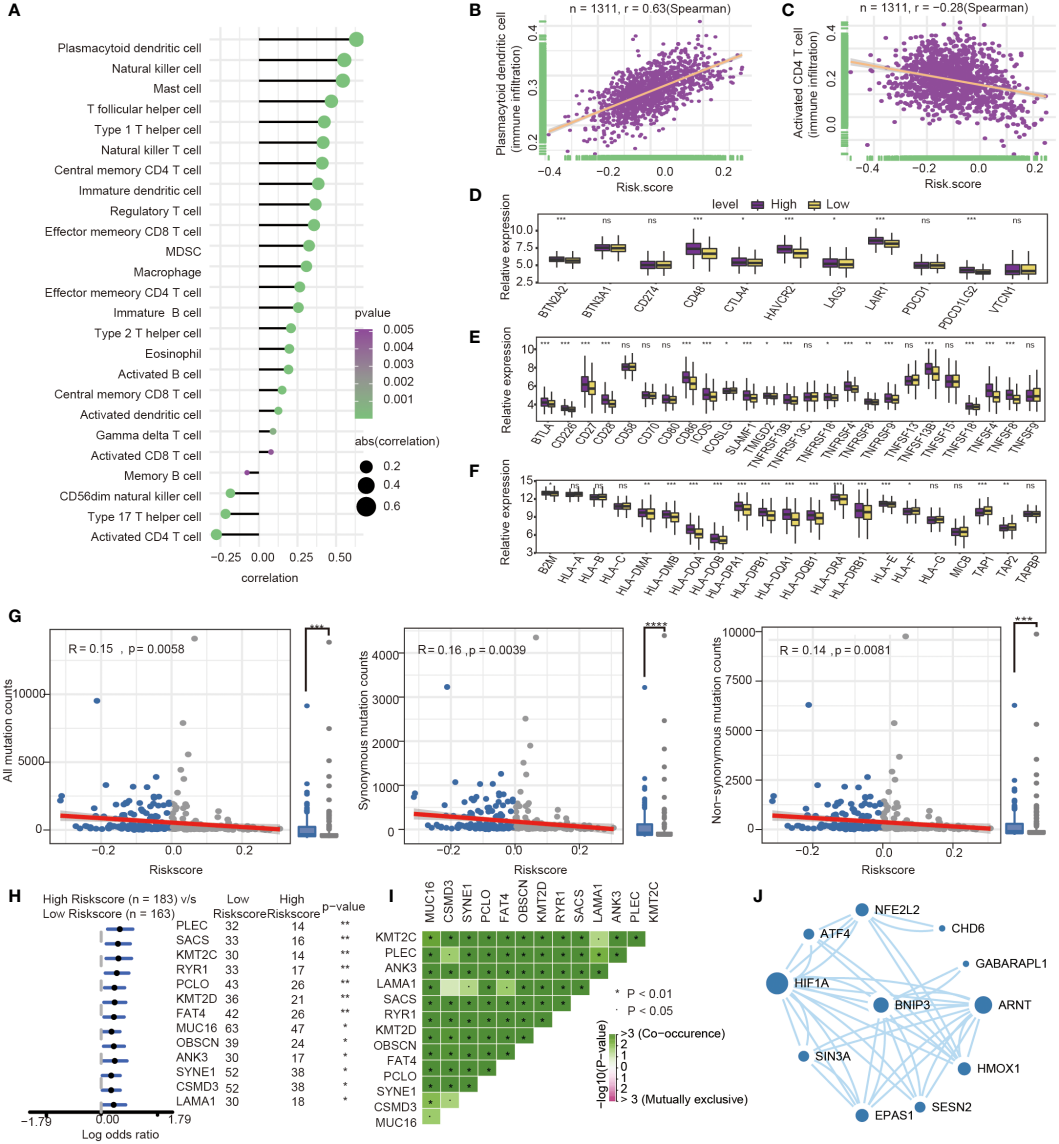
Immune-related characteristics analysis between high-risk group and low-risk group. **(A)** Correlation between immune infiltration pattern and risk score. **(B)** Plasmacytoid dendritic cell expression positively correlated with risk score in GC patients. **(C)** Activated CD4 T cell expression negatively correlated with risk score in GC patients. **(D–F)** The differential expression of immune checkpoint molecules between high-and low-risk groups. (*, P< 0.05; **, P< 0.01; ***, P< 0.001; NS or ns, P > 0.05) **(G)** Association with all mutation counts, non-synonymous mutation counts, synonymous mutation counts in different risk groups. **(H)** Forest plot of genes mutation in GC patients of the low- and the high-risk groups. **(I)** Interaction effect of genes mutating differentially between low- and high-risk groups. **(J)** PPI network for protein of differentially expressed pathways and differential expression genes, the size of circle means the degree of each Protein.

The present research revealed a significant association between risk score and infiltration of 25 immune cell types. Of these, 21 immune cells were positively related to risk score (**Figure 7A**), such as plasmacytoid dendritic cell (Spearman r =0.63, P<0.0001). In comparison, four immune cells showed a negative correlation with the risk score, such as activated CD4 T cell (Spearman r = -0.28, P<0.0001) (**Figures 7B, C**). The expression of immune checkpoint molecules was investigated between high-risk and low-risk groups. Most of the immune checkpoint molecules between the high-risk and low-risk groups, including IAP, ICP, MHC, were expressed differently. The results showed that almost all expression of immune checkpoint molecules in the high-risk group was higher than the low-risk group. Among these immune checkpoint molecules, CTLA-4, one of the most critical immune checkpoint molecules, played an important role in immunotherapy. The expression of CTLA-4 was statistically different between high and low-risk groups. (P<0.05) (**Figures 7D–F**).

Furthermore, we investigated the difference of somatic mutations between the high- and low-risk groups in the TCGA GC dataset. The same tendencies were observed when comparing non-synonymous and synonymous mutation frequency between the low- and the high-risk groups (**Figure 7G**). Mutant distribution of the top 13 genes with the highest mutation frequency between high- and low-risk groups were showed in a waterfall plot (**Figure 7H**), including PLEC, SACS, KMT2C, RYR1, PCLO, KMT2D, FAT4, MUC16, OBSCN, ANK3, SYNE1, CSMD3 and LAMA1(**Figure 7H**, **Table S3**). Interestingly, we observed significant co-occurrences of mutations according to pairwise comparisons in these 13 mutated genes. (**Figure 7I**).

Oxidative stress was one of features of tumor microenvironment, which influenced immune cell functions (28). We compared the different expressions of oxidative stress-related pathways between the high- and low-risk groups. The differentially expressed pathway focused on several oxidative stress-related pathways, such as modulation of the frequency, rate or extent of transcription from an RNA polymerase II promoter under oxidative stress (GOBP_REGULATION OF_TRANSCRIPTION_FROM_RNA_POLYMERASE_II_PROMOTER_IN_RESPONSE_TO_OXIDATIVE_STRESS, *P*= 0.039). The protein encoded by prognosis genes and related to differentially expressed pathways was included in the construction of protein−protein interaction (PPI). The HIF-1α, presenting the highest number of interactions, and the prognosis gene BNIP3 play an important role in oxidative stress and autophagy (**Figure 7J**).

**Figure 8:**
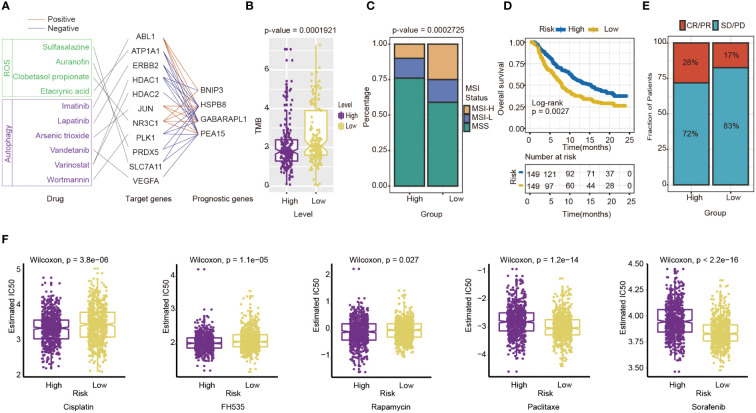
Evaluation of the potential Immunotherapy benefit and prediction chemotherapy sensitivity of different risk groups. **(A)** The interactions between drugs, drug targets and prognostic autophagy-related genes. **(B)** The association between risk score and TMB. **(C)** MSI status differences between high-and low-risk groups. MSI, microsatellite instability; MSI-L, low-level MSI; MSI-H, high-level MSI; MSS, stable MSI. **(D)** Kaplan-Meier survival curves for patients between high- and low-risk groups in IMvigor210 cohort (n=298, P= 0.0027). **(E)** Differences in immunotherapy response between high - and low-risk groups in IMvigor210 cohort CR/PR: response to immunotherapy SD/PD: no response to immunotherapy. **(F)** Chemotherapy sensitivity of different risk groups: from left to right are Cisplatin, FH535, Rapamycin, Paclitaxe and Sorafenib.

Then, some anti-tumor drugs were collected (the information of drug and drug targets were obtained from DurgBank), whose targets were linked with oxidative stress and autophagy. The interactions between prognostic genes and target protein-coding genes are apparent (**Table S4**). For instance, ERBB2, the target of Lapatinib, was negatively associated with the prognostic risk genes, including HSPB8 (*P*=7.155×10^-19^), PEA15(*P*=1.385×10^-10^) and GABARAPL1 (*P*=1.705×10^-24^). ATPA1, the target of Etacrynic Acid, was positively associated with prognostic risk genes, including GABARAPL1(*P*=5.267×10^-13^), HSPB8(*P*=4.102×10^-18^), and PEA15(*P*=4.611×10^-10^) (**Figure 8A**). These results indicated that the effects of oxidative stress-related and autophagy-related drugs might interact with prognostic genes by target protein-coding genes. The results suggested that the prognostic genes are highly related to therapeutic targets, and the four-gene signature may play an important role in immunotherapy and targeted therapy.

The indicators of common biomarkers for predicting the immunotherapy response were also calculated. TMB score of the high-risk group was lower than the low-risk group (Wilcoxon test, *P*= 1.921×10^-5^) (**Figure 8B**). Analysis of microsatellite status suggested that MSI of the low-risk group was higher than the high-risk group (Wilcoxon test, *P*= 2.725×10^-5^) (**Figure 8C**). Finally, the IMvigor210 cohort was applied to predict the results of two groups if they accepted immunotherapy. The patients were divided into a high-risk (n=149) and low-risk group(n=149) by the median of their risk score. The results also showed that the high-risk group had a longer OS than the low-risk group (log-rank test *P*= 0.0027), which preferred to receive immunotherapy (**Figure 8D**). The Chi-square test results showed that the immune response of the high-risk group is higher than the low-risk group (*P*=0.0384) (**Figure 8E**).

### The sensitivity of high-risk and low-risk groups to chemotherapy drugs is different

Next, chemotherapy responses were compared between low-and high-risk groups. In this study, five drugs that affected GC treatment were selected to explore the sensitivity of high and low-risk groups to different chemotherapeutic agents. Drug analysis by pRRophetic R package showed that the low-risk group was more sensitive to Cisplatin (Wilcoxon test, *P*= 3.8×10^-6^), FH535 (Wilcoxon test, *P*= 1.1e−05), and Rapamycin (Wilcoxon test, *P*= 0.027) (**Figure 8F**). In contrast, the high-risk group was more sensitive to Paclitaxel (Wilcoxon test, *P*=1.2×10^-14^) and Sorafenib(Wilcoxon test, *P*<0.0001) (**Figure 8F**). To some extent, these results suggested that the risk score of GC patients could influence the clinical medication regimen.

## Discussion

Overall, we confirmed that there were remarkable differences between clusters of GC based on autophagy-related genes. Then a prognostic signature was constructed based on four autophagy-related genes, and the risk score was calculated by signature, an independent prognostic factor for patients with GC.

Firstly, the clusters based on autophagy-related genes had different molecular characteristics. We found the cell growth and metabolism pathway were enriched in cluster 1, while the EMT pathway was be observed in cluster 2. MYC is a core of the oncogenic process (29) and can promote tumorigenesis with its regulation of transcription (30). E2F plays a crucial role in the CDK-RB-E2F axis, the core transcriptional machinery to drive cell cycle progression (31). E2F2, a member of the E2F family, is a regulator in PI3K/Akt/mTOR pathway, which is strongly associated with autophagy for it inhibits autophagy in GC cells when overexpressed (31). Autophagy is closely connected with the nutrient supply of tumors (32), and PKM2, a critical kinase of glycolysis, promotes cell migration and inhibits autophagy contributing to the malignant development of gastric cancer (33). EMT improves the aggressiveness of cancer cells (34), which influences cancer progress by interaction with autophagy (19). The EMT score also shows the same results between the two clusters, as cluster 2 is more enriched in the EMT pathway. These results provide initial evidence that GC cells show different characteristics when the expression of autophagy-related genes in GC is different in the two clusters. The results of GSVA can hint that the pathogenesis of GC is related to autophagy-related genes expressing to some extent. This work may provide a basis for building new molecular typing methods in GC patients.

In addition, we find several autophagy regulators with superior prognostic value, such as PEA-15, BNIP3, GABARAPL1, and HSPB8. These autophagy-related genes are associated with cancer initiation and progression in many aspects. PEA-15, known as phosphoprotein enriched in diabetes (PED) (35), is connected with cell survival and glucose metabolism (36). PEA-15 is related to the drug resistance of cisplatin, the first-line chemotherapeutic drug gastric cancer (37). BNIP3 is one of the mitophagy receptors playing a suppressing role in breast cancer (38). In gastric cancer, tumor cells partly occur aberrant methylation of BNIP3 but not in adjacent normal tissues, which indicates that inactivation of BNIP3 would promote gastric cancer progress (39). Hypoxia provokes oxidative stress, as it is accompanied by increasing ROS production (40). HIF-1α is the principal regulator of hypoxia (41), and is also led to autophagy activation with BNIP3 in breast cancer (42). Knockdown of GABARAPL1, an early estrogen-regulated gene belonging to the GABARAP family (43), inhibits AR-positive prostate cancer growth. Correspondingly, GABARAPL1 has been reported to promote tumor growth by increasing FL-AR/AR-V transcription activity (44). HSPB8 is one of the heat shock protein families. In breast cancer, E2 could activate HSPB8, which promotes breast tumor cells growth by MAPK Signaling (45).

In the current research, the four prognostic autophagy-related genes are applied to the constructed prognostic signature, and the risk scores of patients with GC are calculated with this signature. Previously, there are some prognostic signatures based on GC’s autophagy-related genes (46), such as autophagy-clinical prognostic index (47) and a six-gene-based prognostic model (48). The signatures above only reveal a predictive power, while our four-gene prognostic signature can predict the prognosis, biomarkers in immunotherapy, and therapy response. The signature we constructed provides more comprehensive prognostic information for patients with GC. From this study, a risk score based on autophagy-genes is one of the prognostic factors of GC. The risk score is associated with clinical-pathological factors, including age and stage. Autophagy decreases with age, and age-related diseases are induced by impaired autophagy predisposes (49). And the stage is a major factor in treatment method determining and patient prognosis predicting (50). So, the risk score was proven to be a robust model for GC patient outcome prediction. Nomogram is a usual method to estimate prognosis in oncology, which is more exact and user-friendly than conventional stage (51). Nomogram is widely used in the prognosis of GC, such as predicting gastric cancer recurrence by biomarker gene expression (52), predict the risk of peritoneal metastasis in GC with serosal invasion after radical surgery (53), and so on. The nomogram integrated risk score with various clinical parameters in our study, intuitively highlighting their weight in GC prognosis.

Furthermore, the risk score of patients with GC has potential application to predicting therapy response. Now, immunotherapy is applied in gastric cancer treatment, especially late-stage metastatic gastric cancer (9). Plasmacytoid dendritic cells, positively correlated with the presently constructed risk score, contribute to tumor immunologic tolerance rather than anti-effect (54). The activated CD4 T cell is negatively correlated with the risk score and may facilitate cancer immunotherapy by improving cytotoxic T cell response (55). Immune checkpoints molecules, including anti-cytotoxic T lymphocyte antigen-4 (CTLA-4), anti-programmed cell death-1 (PD-1), and anti-programmed cell death ligand-1 (PD-L1), are the thoroughly investigated class of immunotherapy (56, 57). TMB and MSI, independent of expression of PD-L1, are widely applied biomarkers in immunotherapy of cancer (58). GC patients with high MSI characteristic shows positive outcoming in most research regardless the disease stage (20). Compared with low TMB patients, high TMB patients show significantly better responses and longer survival advantage in many cancers such as non-small-cell lung cancers (59). High TMB may be a predictive marker of advanced gastric cancer received toripalimab (one type of immunotherapy drug) (60). Risk score has a relatively good correlation with a prognostic indicator of response to immunotherapy above. It can be a comprehensive indicator for decisions regarding immunotherapy in GC patients. The result of IMvigor210 cohort suggest that high-risk group has better prognosis with immunotherapy, that is in contrast to expectations in training and validation cohorts. Cancers are heterogeneous, even so, risk score absolutely has the ability to divide cancer patients into high-and low-risk groups with survival significance statistically.

Chemotherapy has been an important part of the treatment of gastric cancer. Based on the grouping in risk score, the sensitivity of drugs between the two groups is different. Cisplatin and paclitaxel, the drugs enrolled in this study, are first-line chemotherapy of gastric cancer, and they are usually on behalf of varying treatment projects. In the Chinese society of Clinical Oncology (CSCO), for metastatic gastric cancer, cisplatin is suitable for HER2 positive patients, and paclitaxel is available for HER2 negative patients (9). Besides above, Sorafenib plays an anti-tumor role by inhibiting gastric cell growth and induces apoptosis combined with cisplatin (61). FH535, the small molecule inhibitor of canonical wnt signaling (62), can inhibit tumor proliferation and moderate invasion of gastric cancer (63). The specific mTOR inhibitor, Rapamycin, can treat gastric cancer combined with other chemotherapy drugs (64). Interestingly, mTOR is a pharmacologic target of autophagy (65), and the exploration of chemotherapy drugs of gastric cancer can continue from autophagy by mTOR. Above all, the risk score may be a direction to estimate the risk of GC patients and ensure proper clinical medication for patients.

Despite the strengths associated with our study, there were also some limitations. We utilized several large GC datasets containing over 1000 samples. However, more GC samples are required to verify the reliability of our conclusion. Another drawback is that the cohort of immunotherapy-therapied GC patients is not available now. We look forward to obtaining more immunotherapy clinical cohorts with GC to verify the risk score in an actual treatment environment. It might enable the selection of appropriate patients for individual f therapeutic regimens. Finally, the function of the autophagy-related genes requires more experimental verification.

In conclusion, these findings have significant implications for the understanding of autophagy-related genes in GC. The risk score based on autophagy-related genes could serve as a potential prognostic biomarker. It will be significant for guiding therapeutic strategies for GC for there is a prominent association between risk score and treatment response.

## Data availability statement

The original contributions presented in the study are included in the article/**Supplementary Material** and publicly available. Further inquiries can be directed to the corresponding author.

## Ethics statement

Ethical approval was not required for this project as all data were taken from the public domain.

## Author contributions

BL and KC were responsible for study design, critical revision of the manuscript and obtaining funding. YY wrote and revised the manuscript. JM, XH, YT and LW analyzed data, interpreted results, and helped to write the manuscript. All authors contributed to the article and approved the submitted version.

## Supplementary material

Supplementary Table 1 The baseline information of the patients.

Supplementary Table 2 The results of Subgroup and risk score of GC samples.

Supplementary Table 3 The OR of deferentially mutating genes in patients of the low- and the high-risk groups.

Supplementary Table 4 The expression correlation of target protein-coding genes and prognostic genes, assessed using Pearson correlation analysis.

Supplementary Text Including materials and methods, **Figures S1**, **S2**.
