# Supplementary material for: Comprehensive analysis of autophagy-related clusters and individual risk model for immunotherapy response prediction in gastric cancer
Source: Front Oncol. 2023 Mar 3;13:1105778. doi: 10.3389/fonc.2023.1105778 (PMC10022822; doi:10.3389/fonc.2023.1105778)
Supplement: Supplementary Text — Including materials and methods, Figures S1 , S2 . [file DataSheet_1.pdf]

Supplementary Material

Comprehensive analysis of autophagy-related clusters and individual risk model for immunotherapy response prediction in gastric cancer

Yanxin Yao[1#], Xin Hu[1#], Junfu Ma[1#], Liuxing Wu, Ye Tian, Kexin Chen, Ben Liu[*]

*** Correspondence:** Ben Liu: benliu100@tmu.edu.cn

1 Supplementary Data

Materials and methods

Gastric cancer dataset source and preprocessing

The RNA-Seq profile and clinical information of GC patients were downloaded from the TCGA database (http://www.cancergenome.nih.gov/), and the expression profile and clinical information of four gastric cancer cohorts, including GSE15459, GSE34942, GSE62254/ACRG, GSE84437, GSE13861, GSE28541, GSE13861, and GSE28541 were acquired from the GEO database (https://www.ncbi.nlm.nih.gov/geo/). TCGA and GSE15459, GSE34942, GSE62254/ACRG, GSE84437, GSE13861, and GSE28541 datasets were merged and enrolled in this study as the training set. GSE13861, and GSE28541 were served as validation set. Batch effects of these cohorts enrolled were removed by sva R package. The autophagy-related genes were obtained from the HADb website (http://www.autophagy.lu/). The data of TMB, MSI, and mutation genes were obtained from TGCA and GEO databases.The autophagy-related genes were obtained from the HADb website (http://www.autophagy.lu/).

Construction of prognostic signature and risk score calculation

Univariate Cox regression analysis was used based on selected DEGs, and P<0.05 was considered confident in statistics. Following this analysis, LASSO regression was used to further select prognostic genes with glmnet R package.

Analysis of subtypes biological features

Gene set variation analysis (GSVA) was operated by GVSA R package to analyzed pathway enrichment degrees between different subtypes. EMT scores(1) were calculated by GSVA R package. The MutationalPatterns R package was used to compared mutation landscape and mutation signature between different subtypes. A total of 30 mutation signatures were obtained from the COSMIC website (https://cancer.sanger.ac.uk/cosmic/signatures). Somatic mutations were evaluated by VanScan2, and copy number variation (CNV) was assessed by GISTIC 2.0 method. The waterfall plot of a mutational landscape was generated using GenVis R package. CNV databases were downloaded from TCGA website (http://portals.broadinstitute.org/tcga/home), and the location and frequency of copy number variation of DEGs are also analyzed.

Evaluation of prognostic signature

Functional enrichment analysis was performed by the Metascape webtool (http://metascape.org)(2). Oxidative stress-related pathways were collected from Gene Set Enrichment Analysis (https://www.gsea-msigdb.org/gsea/), and we focused on oxidative stress-related pathways performing Gene Ontology (GO) analysis.

The survival curves were drawn by the Kaplan-Meier plot. The difference in the survival status between the high- and low-risk groups was assessed. The Tianjin cohort was applied to verify the validity and robustness of the prognosis signature. Then, multivariate Cox regression analysis was used to estimate the relationship between clinical-pathological factors and risk scores. Finally, based on the identified prognostic factors, the nomogram was constructed with nomogramEx R package for predicting 3-and 5- survival rates of GC patients. Oxidative stress related pathways were enriched for significant differential expression, using STRING (https://www.string-db.org/) and Cytoscape to analyze protein interaction study.

Immunotherapeutic and Chemotherapeutic response prediction with prognostic signature

Major histocompatibility complex (MHC), immune co-inhibitory checkpoints (IAP), and immune co-stimulator checkpoints (ICP) were used to assess the expression level of immune checkpoint molecules between two groups. Besides, TMB and MSI were estimated between high-risk and low-risk groups with a Chi-square test. IMvigor210 cohort (http://research-pub.gene.com/IMvigor210CoreBiologies)(3). 21 Differentially Expressed Genes were used to validate the efficiency of prognostic risk signatures with immunotherapy. The R package pRRophetic was operated to analyze the sensitivity of chemotherapy drugs. Several autophagy- and oxidative stress-related drugs and their targets were obtained from DrugBank (https://go.drugbank.com/). The correlation between target protein-coding genes and prognostic genes was measured using the Pearson correlation statistic.

Statistical analysis

OS time differences between different groups were calculated by K-M survival analysis. The Pearson correlation test analyzed the correlation between molecules. Receiver operating characteristic curve (ROC)was applied to assess specificity and sensitivity by R package “pROC”.

Figure S1

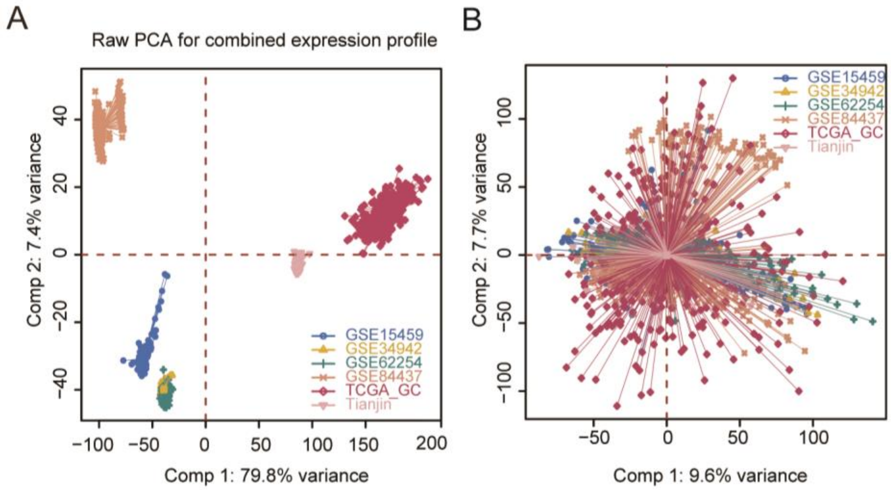

FigureS1 Removal of batch effect.

After applying the batch-correction methods, the batch effects among 6 gene sets were all eliminated to some extent**.**

A Samples from 6 data sets before batch correction. **B** Samples from 6 data sets after batch correction.

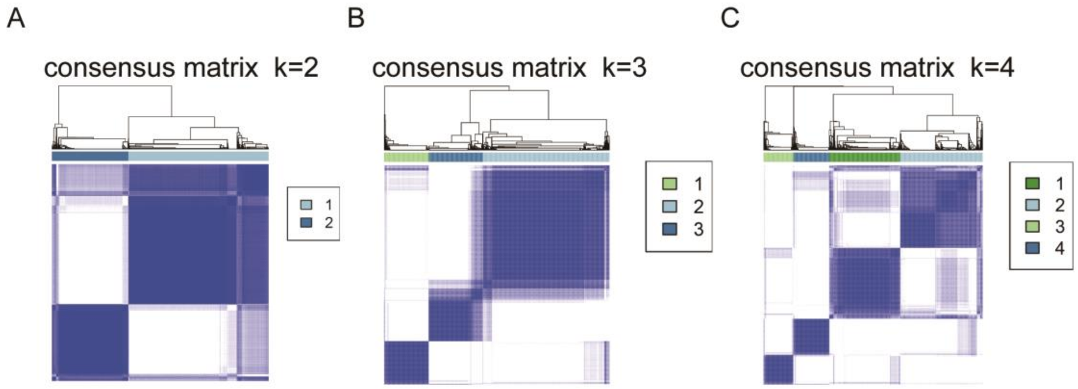

Figure S2

FigureS2 Consensus clustering matrix of 1311 GC samples. A k = 2 **B** k=3 **C** k=4

1. Tao C, Huang K, Shi J, Hu Q, Li K, Zhu X. Genomics and Prognosis Analysis of Epithelial-Mesenchymal Transition in Glioma. *Front Oncol* (2020) 10:183. Epub 2020/03/11. doi: 10.3389/fonc.2020.00183.

2. Zhou Y, Zhou B, Pache L, Chang M, Khodabakhshi AH, Tanaseichuk O, et al. Metascape Provides a Biologist-Oriented Resource for the Analysis of Systems-Level Datasets. *Nat Commun* (2019) 10(1):1523. doi: 10.1038/s41467-019-09234-6.

3. Tauriello DVF, Palomo-Ponce S, Stork D, Berenguer-Llergo A, Badia-Ramentol J, Iglesias M, et al. Tgfβ Drives Immune Evasion in Genetically Reconstituted Colon Cancer Metastasis. *Nature* (2018) 554(7693):538-43. doi: 10.1038/nature25492.
